# Supplementary material for: Miniaturized electromechanical devices with multi-vibration modes achieved by orderly stacked structure with piezoelectric strain units
Source: Nat Commun. 2022 Nov 2;13:6567. doi: 10.1038/s41467-022-34231-7 (PMC9630418; doi:10.1038/s41467-022-34231-7)
Supplement: Supplementary file 1 — Supplementary Information [file 41467_2022_34231_MOESM1_ESM.pdf]

Supplementary Information

Miniaturized electromechanical devices with multi-vibration modes achieved by orderly stacked structure with piezoelectric strain units

Jinfeng Liu[1], Xiangyu Gao[1*], Haonan Jin[1], Kaile Ren[1], Jingyu Guo[1], Liao Qiao[1], Chaorui Qiu[1], Wei Chen[2], Yuhang He[2], Shuxiang Dong[3,4], Zhuo Xu[1], Fei Li[1*]

[1] Electronic Materials Research Laboratory, Key Lab of Education Ministry and State Key Laboratory for Mechanical Behavior of Materials, School of Electronic Science and Engineering, Xi'an Jiaotong University, Xi'an 710049, China.

[2] OPPO Guangdong Mobile Communication Co., LTD. Shenzhen 518051, China

[3] School of Materials Science and Engineering, Peking University, Beijing 100871, China

[4] Institute for Advanced Study, Shenzhen University, Shenzhen 518051, China

*Corresponding author: gaoxiangyu@xjtu.edu.cn; ful5@xjtu.edu.cn

The PDF file includes:

Supplementary Note 1. Designing of the orderly stacked structure with piezoelectric strain units (OSSPSU). 2

Supplementary Fig. 1 2

Supplementary Note 2. Piezoelectric properties and driving principle of the [001]-PIMNT crystal and hard ceramic-OSSPSU stators. 4

Supplementary Fig. 2 4

Supplementary Table 1. 5

Supplementary Fig. 3 6

Supplementary Note 3. The frequency dependence of the displacements of the OSSPSU stator 7

Supplementary Fig. 4 8

Supplementary Note 4. Effect of external force on the properties of OSSPSU stator 9

Supplementary Fig. 5 9

Supplementary Note 5. Theoretical derivation of motion velocity 10

Supplementary Fig. 6 11

Supplementary Note 6. The step resolution measurement of the micro liner ultrasonic OSSPSU motors.12

Supplementary Fig. 7 13

Supplementary Fig. 8 14

Supplementary Fig. 9 14

Supplementary Table 2. 15

Supplementary Note 7. The application of the micro linear ultrasonic OSSPSU motor. 16

Supplementary Fig. 10 16

Supplementary Fig. 11 17

Supplementary Note 8. Miniature piezoelectric OSSPSU motor stators and their experimental setup. . 18

Supplementary Fig. 12. 18

Supplementary References 19

Supplementary Note 1. Designing of the orderly stacked structure with piezoelectric strain units (OSSPSU).

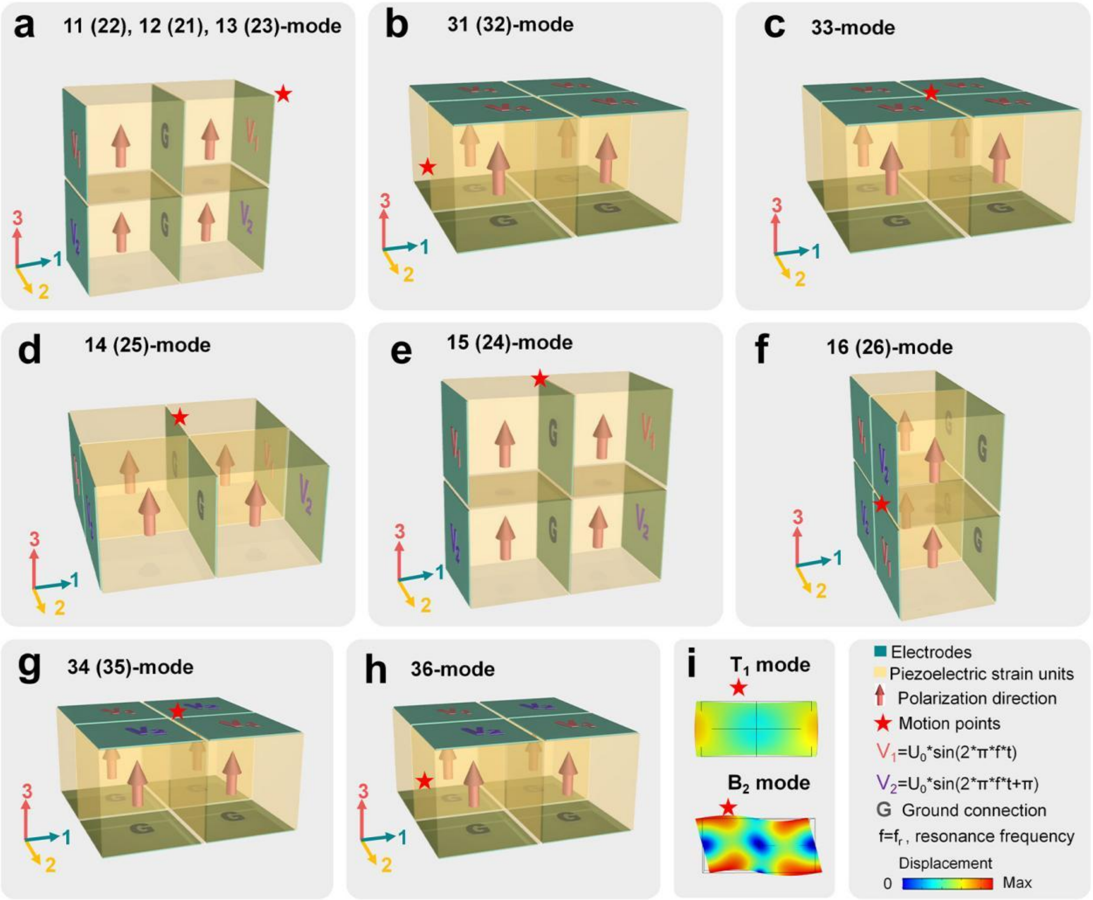

Supplementary Fig. 1 Design of the four-unit OSSPSU. a, The applied electric voltage signals and deformation schematics of artificial 11 (22)-, 12 (21)- and 13 (31)- modes; **b**, artificial 31 (32)- mode; **c**, artificial 33-mode; **d**, artificial 14 (25)-mode; **e**, artificial 15 (24)-mode; **f**, artificial 16 (26)-mode; **g**, artificial 34 (35)-mode; **h**, artificial 36-mode at resonance frequencies. **i,** The simulated deformations diagrams of **a**-**h**.

As shown in Figs. 3a-b, the normal strain and shear strain can be realized by exciting the T_1 mode and B_2 mode of the OSSPSU, respectively. Theoretically, the strain ε_λ of OSSPSU generating normal vibration modes can be estimated by the following formula:

$$\varepsilon_\lambda = \varepsilon_{ij} = \frac{\delta L_j}{L_j}, (\lambda = i = j = 1,2,3) \tag{S1}$$

Where L_j is the outline dimension of the OSSPSU; δL_j is the displacement along j-direction of the motion points P_L (point on the left of the selected deformation region), C (point on the center of the selected deformation region), or P_R (point on the right of the selected deformation region). According to the simulation results, the shear strain ε_λ of OSSPSU will be excited via synergistic effect in i-j plane. The strain ε_λ of OSSPSU generating shear vibration modes can be computed as:

$$\varepsilon_\lambda = \varepsilon_{ij} = \theta_{ij} + \theta_{ji} = \frac{|\delta L_j|}{|l_i + \delta l_i|} + \frac{|\delta l_i + \delta l_i'|}{|L_j - \delta L_j' + \delta L_j|} \approx \frac{|\delta L_j|}{l_i} + \frac{|\delta l_i|}{L_j}, \quad (\lambda = 9 - i - j, i \neq j, i = 1,2,3 \ j = 1,2,3) \tag{S2}$$

Where l_i is the outline dimension of the shear strain deformation region of the OSSPSU; δl_i and $\delta l_i'$ are the displacements along i-direction of P_R and P_R', respectively; $\delta L_j'$ is the displacement along j-direction of P_R'; θ_{ij} and θ_{ji} are the shear angles in i-j plane shown in Fig. 3b. The above formulas are also applicable to the calculation of the strains of coupled vibration modes.

According to the Euler-Bernoulli beam model, the resonant frequency of the first transverse (T_1) mode and the second bending (B_2) mode of the cuboid piezoelectric unit can be approximately estimated[1]

$$f_{T_1} = \frac{1}{2L_L}\sqrt{\frac{1}{\rho s_{11}^E}} \tag{S3}$$

$$f_{B_2} = \frac{a^2 L_W}{4\pi L_L^2 \sqrt{3}}\sqrt{\frac{1}{\rho s_{11}^E}} \tag{S4}$$

where L_L, L_W, and ρ are the length, width and density of the piezoelectric material, respectively; s_{11}^E is the elastic compliance constant of the material; a is a constant equal to 7.853 derived from the Euler-Bernoulli beam model.

Theoretically, the resonance frequencies of T_1 and B_2 modes have no connection with the height (L_H) of the piezoelectric material. In order to make the values comparable, the simulation conditions need to keep the consistency, thus, the height-to-length ratio (HLR) of the piezoelectric units is selected for simulation calculation of the ε_λ.

Supplementary Note 2. Piezoelectric properties and driving principle of the [001]-PIMNT crystal and hard ceramic-OSSPSU stators.

To effectively excite the diagonal liner motion of the friction tip, the resonant frequencies (f_r) of the 31-mode and the 36-mode should be as close as possible. Therefore, we used the FEM to perform modal simulation and size optimization of the piezoelectric OSSPSU stator. Considering the miniaturization design, we fixed the length (L_L) of the piezoelectric stator as 5 mm, and the height (L_H) as 1.3 mm. The friction tip size was fixed as φ0.8 mm^l × 0.5 mm^h. Through FEM simulation, the dependence of the two vibration modes on the width (L_W) was obtained.

As shown in Supplementary Fig. 2a, with the increase of L_W, the f_{31} of the [001]-PIMNT crystal-OSSPSU stator gradually decreases and the f_{36} gradually increases, and the two curves intersect at L_W=1.06 mm. To make the two resonant frequencies as close as possible, the optimized single crystal-OSSPSU stator size is 5 mm^{Length} × 1.3mm^{Height} × 1.06 mm^{Width}. At this time, the f_{31} and f_{36} are 163.13 kHz and 163.3 kHz, respectively. Supplementary Fig. 2b shows the variation of two resonant frequencies of hard ceramic-OSSPSU stator with respect to W. As the height W increases, both resonant frequencies decrease, and the f_{36} decreases slightly faster than f_{31}, so they intersect at L_W=3.24 mm. After optimization, the size of the ceramic-OSSPSU stator is set to be 5 mm^{Length} × 1.3mm^{Height} × 1.06 mm^{Width}. The resonance frequencies of f_{31} and f_{36} are 327.51 kHz and 327.34 kHz, respectively. Obviously, the volume of the ceramic-OSSPSU stator is three times that of the single crystal stator (Supplementary Fig. 2c).

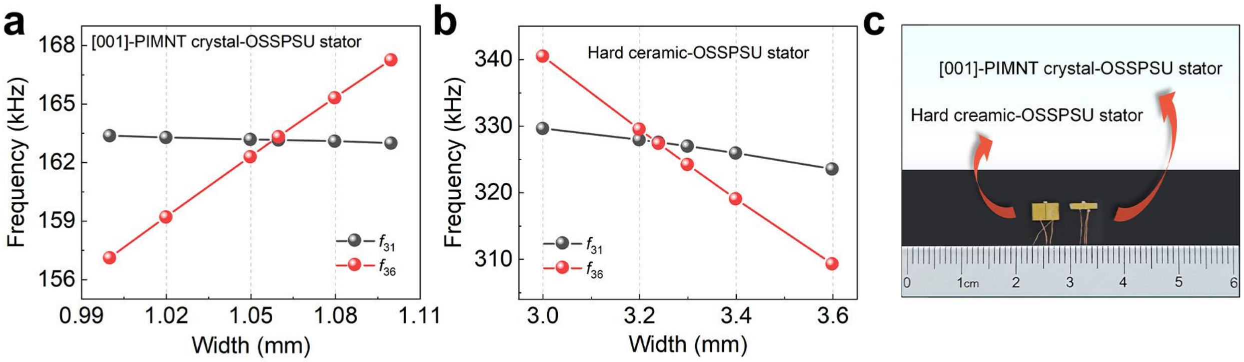

Supplementary Fig. 2 FEM simulation of the ultrasonic OSSPSU stators. a-b, Frequencies of the 31- and 36-modes change with different dimension of L_W *(width)* for [001]-PIMNT single crystal and hard ceramic-OSSPSU stators, respectively. **c**, Photographs of the piezoelectric OSSPSU stators.

The electromechanical coupling coefficient (k) is an important parameter used to express the mutual conversion ability of mechanical energy and electrical energy in a piezoelectric stator. Herein, the effective electromechanical coupling coefficient (k_{eff}) of the two OSSPSU stators can be calculated from Fig. 4b-c as follows[2]:

$$k_{eff}{}^{2} = 1 - \frac{f_{\mathrm{r}}^{2}}{f_{\mathrm{a}}^{2}} \tag{S5}$$

As listed in Table 1, from the calculation results of experimental and simulation data (calculated by Supplementary Eq. (S5)), due to the larger bandwidth, the k_{eff} of the crystal-OSSPSU stator is about twice that of the ceramic-OSSPSU stator, indicating that the former may have more significant advantages in output efficiency as a piezoelectric motor.

Supplementary Table 1. The effective electromechanical coupling factor k_{eff} of [001]-PIMNT crystal and hard ceramic piezoelectric OSSPSU stators calculated by experiment and FEM simulation data.

Piezoelectric stators	Experimental			Simulated		
	f_{r} (kHz)	f_{a} (kHz)	k_{eff} (%)	f_{r} (kHz)	f_{a} (kHz)	k_{eff} (%)
[001]-PIMNT crystal-OSSPSU stator	168.3	175.6	28.5	163.0	169.5	27.4
Hard ceramic OSSPSU-stator	330.3	333.6	14.0	327.5	330.0	12.3

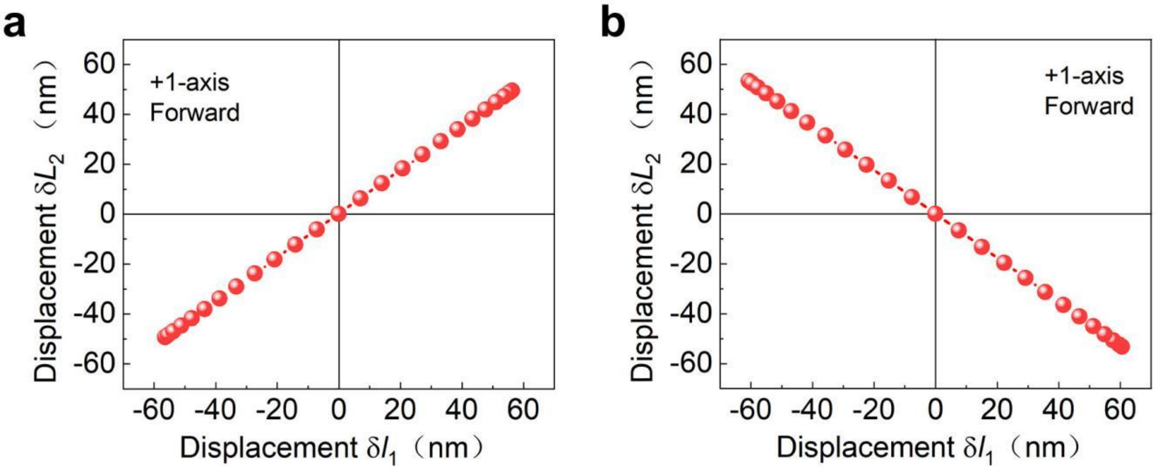

Supplementary Fig. 3 Motion trajectories of the friction tip and the crystal-OSSPSU stator. a-b, Motion trajectory for the linear driving in the +1-direction and -1-direction, respectively.

Supplementary Note 3. The frequency dependence of the displacements of the OSSPSU stator

The variation of the coupled horizontal (δl_1, along the 1-axis, as shown in Fig. 4a) and the coupled vertical (δL_2, along the 2-axis, as shown in Fig. 4a) displacements of the friction tips for the piezoelectric OSSPSU stators were tested as a function of frequency (Supplementary Fig. 4) under the electric field intensity of 20 V mm^{-1}. Obviously, the operating frequency range of the crystal-OSSPSU stator is wider than that of the ceramic-OSSPSU stator, owing to the larger bandwidth (shown in Fig. 4b-c). Moreover, the slight difference between two channels is due to the minor errors in fabrication of the piezoelectric OSSPSU stators[3]. As shown in Supplementary Fig. 4a and b, the resonant frequencies of the crystal-OSSPSU stator along the 1-axis and 2-axis are around 167 kHz, and the maximum displacements are around 80 nm and 75 nm, respectively. For the ceramic-OSSPSU stator, the resonant frequencies along the 1-axis and 2-axis are all around 332 kHz, and the maximum displacements are around 45 nm and 65 nm, respectively (Supplementary Fig. 4c and d). Comparing the data in Supplementary Fig. 4, the amplitude of the crystal-OSSPSU stator along 2-axis is relatively close, but the the amplitude of the crystal-OSSPSU stator along 1-axis is 1.7 times that of the ceramic-OSSPSU stator. In addition, according to Eq. (4), it can be calculated that the strain ε_6 of the artificial 31-36 vibration mode of the crystal and ceramics-OSSPSU stators are around 0.026% and 0.018%, respectively, much higher than that of natural piezoelectric materials. Furthermore, there is a certain difference between the test value (0.026%) and the simulation value (0.039%) of the ε_6 of 31-36 coupled mode of crystal stator under the same electric field, which may be caused by the difference in boundary conditions and mechanical losses between the simulation model and the experimental model.

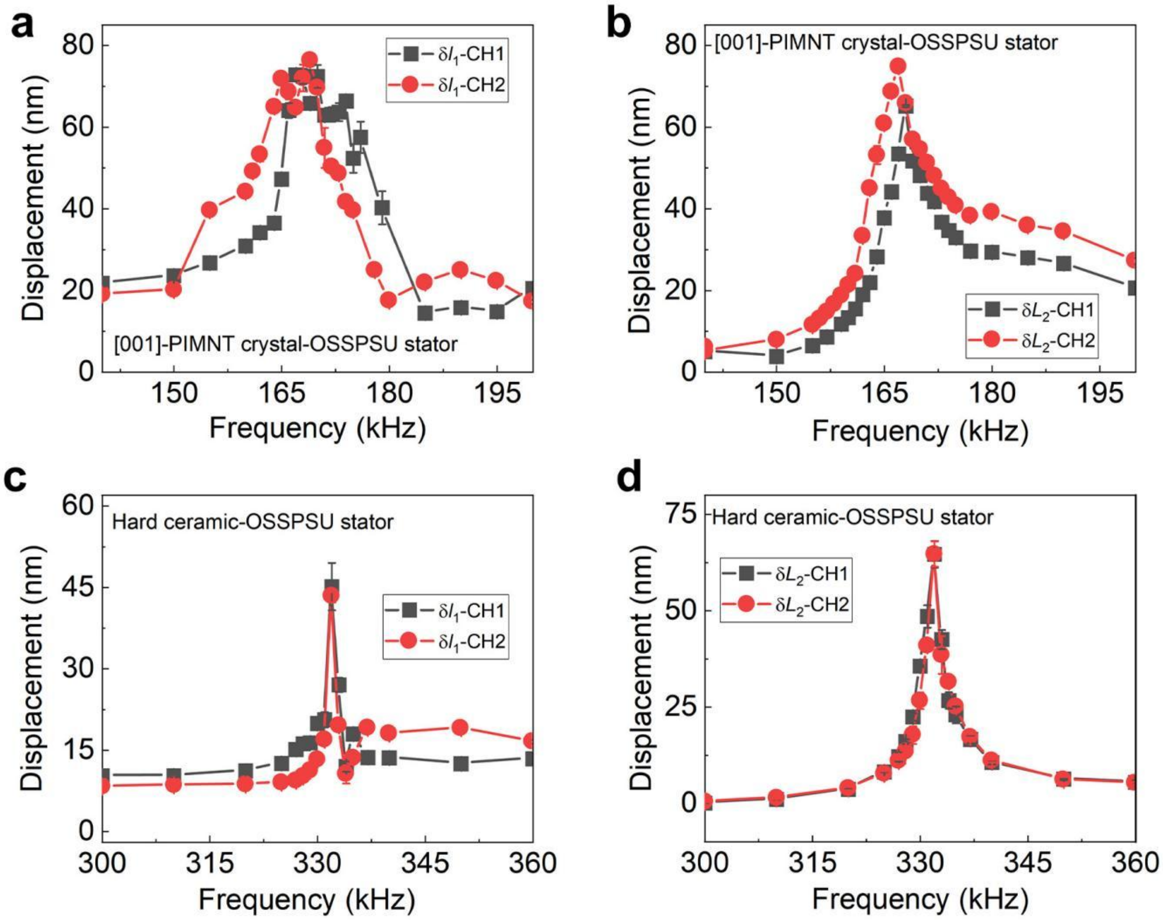

Supplementary Fig. 4 The frequency dependence of the displacements of the friction tips. a-b, The coupled horizontal (δl_1, along the 1-axis) and the coupled vertical (δL_2, along the 2-axis) displacements of the friction tip for the [001]-PIMNT crystal-OSSPSU stator; **c-d**, hard ceramic-OSSPSU stator under the electric field of 20 V mm^{-1}. The error bar shows the standard deviation (three times are measured for each point).

Supplementary Note 4. Effect of external force on the properties of OSSPSU stator

We investigated the effect of uniaxial stress on the impedance spectra of 31-36 coupled mode OSSPSU stators by a home-made uniaxial stress loading setup[4]. The stress dependence of impedance spectra of OSSPSU stators were measured by the LCR meter (Agilent E4294A). During the test, the uniaxial stress was applied perpendicular to the polarization direction, as shown in Supplementary Fig. 5a. Supplementary Figs. 5b-c show the test results of the [001]-PIMNT crystal and hard ceramic piezoelectric OSSPSU stators. It can be seen that, with increasing the uniaxial stress, the intensity of both resonance and anti-resonance peaks in impedance curves decrease, which become more diffuse and shift towards higher frequencies.

Furthermore, with the increase of uniaxial stress, the impedance spectra of the [001]-PIMNT crystal and hard ceramic piezoelectric OSSPSU stators tend to split. This phenomenon shows that the OSSPSU stator is possible to be decoupled. Therefore, for electromechanical devices under the working condition with large uniaxial stress, the preload stress should be considered for the OSSPSU design.

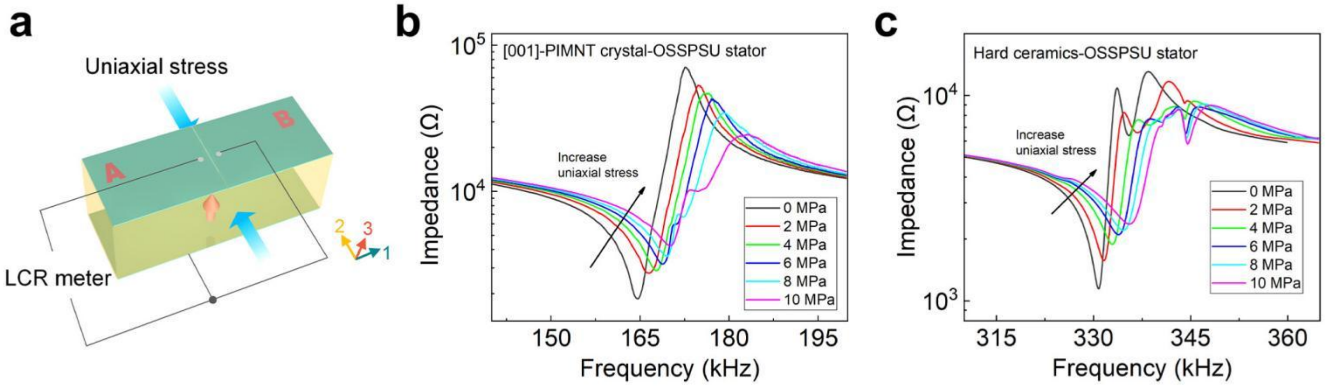

Supplementary Fig. 5 The schematic figure of OSSPSU stator, and the uniaxial stress dependence of impedance spectra of 31-36 coupled mode OSSPSU stators. a, The schematic figure of OSSPSU stator (red arrows represent polarization direction, and blue arrows represent the applied uniaxial stress). **b**, The impedance of [001]-PIMNT crystal-OSSPSU stator measured under uniaxial stress. **c**, The impedance of the hard ceramic-OSSPSU stator measured under uniaxial stress.

Supplementary Note 5. Theoretical derivation of motion velocity

To experimentally verify the actual performance of the OSSPSU stator (with the size of 5 mm^{Length} × 1.3mm^{Height} × 1.06 mm^{Width}), we proposed a linear ultrasonic OSSPSU motor as shown in Fig. 5a. Which consists of a base, a piezoelectric OSSPSU stator with a friction tip (zirconia, with the size of φ0.8 mm^{l} × 0.5 mm^{h}), a stator holder, a slider with a friction plate made of zirconia, and a preloading structure composed of springs and screw. When a sinusoidal alternating current (AC) voltage excitation signal ($\sin(2\pi ft)$) was applied to CH1 or CH2, the friction tip forms the continuous microscopic oblique liner motion, which in turn pushes the slider to perform macroscopic linear motion through friction force. To describe the output properties of the piezoelectric ultrasonic motor, the OSSPSU stator is considered to be a Timoshenko beam[5,6]. When the friction tip is in contact with the friction plate, the motion velocity of the slider (v_s) along 1-axis can be expressed as

$$\frac{\partial v_s}{\partial t} = \frac{F_T + F_f + F_1}{M} \tag{S6}$$

with

$$F_f = \mu_s (F_N + Mg) \tag{S7}$$

$$F_T = \mu_p F_N \tag{S8}$$

where t is the motion time, M is the mass of slider, μ_s, F_f, μ_p, F_T are the friction coefficient and the friction force of the liner guide of the slider, and between the friction tip and the friction plate, respectively. $F_N = F_P + k_e \delta L_2$ is the normal force in 2-axis when the friction tip is in contact with the friction plate, where F_P is the applied preload force, k_e is the equivalent contact stiffness of the contact tip, and δL_2 is the maximum displacement of the friction tip along the 2-axis. $\delta L_2^{'} = \delta L_2 \sin(2\pi ft)$ is the total displacement. Where f is the resonant frequency, t is the time of vibration.

Furthermore, $F_1 = k_e \delta l_1^{'}$, where $\delta l_1^{'} = \delta l_1 \sin(2\pi ft)$ is the total displacement of the friction tip along 1-axis, and δl_1 is the maximum displacement along 1-axis. Combined with Supplementary Eqs. (S2), and (S6)-(S8), the v_s can be calculated as follows:

$$v_s = \int \frac{(\mu_p + \mu_s)[F_p + k_e \delta L_2 \sin(2\pi ft)] + k_e \delta l_1 \sin(2\pi ft) + \mu_s Mg}{M} dt$$
$$= \int \frac{(\mu_p + \mu_s)[F_p + k_e \theta_{12} (l/2) \sin(2\pi ft)] + k_e \theta_{21} L_W \sin(2\pi ft) + \mu_s Mg}{M} dt \qquad \text{(S9)}$$

Where θ_{12} and θ_{21} are the shear angles on the plane perpendicular to the 3-axis at 31-36 coupled vibration mode.

Therefore, the slider motion speed is closely related to the stator piezoelectric performance, the copuled displacement along 1-axis (δl_1) and 2-axis (δL_2) (i.e., the strains of the artificial 31-36 vibration mode), preload force, friction coefficient, and slider mass.

To determine to the optimal preload force of the OSSPSU stator, we designed a home-made test setup, as shown in Supplementary Fig. 6. By this test setup, the optimal preload force of PIMNT crystal-OSSPSU stator is found to be about 300 mN.

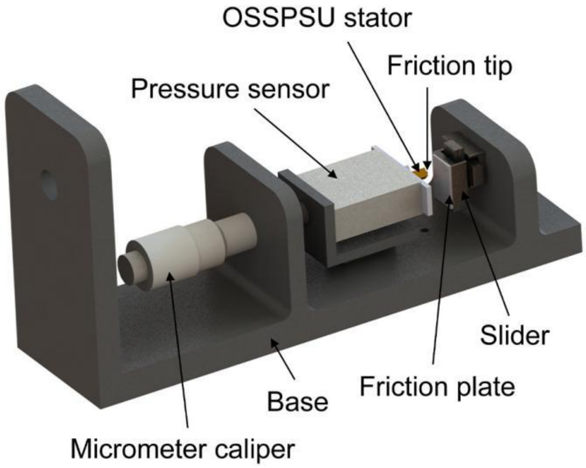

Supplementary Fig. 6 Home-made preload force test setup of the OSSPSU stator.

Supplementary Note 6. The step resolution measurement of the micro liner ultrasonic OSSPSU motors.

In addition, the detailed bidirectional motion steps of the OSSPSU motors under different number of period of pulse signals are shown in Supplementary Fig. 7.

Combined with Supplementary Figs. 8a-b, under the electric field of 20 V mm^{-1}, the transient displacement response of the coupled horizontal (δl_1 along the 1-axis) and coupled vertical (δL_2 along the 2-axis) directions of the friction tip for [001]-PIMNT crystal-OSSPSU stator gradually increases in the first few cycles, and then stabilizes at a higher level (which corresponds to the values in Supplementary Fig. 4a-b), while the ceramic-OSSPSU stator (Supplementary Fig. 8c-d) stabilizes at a high level (which corresponds to the values in Supplementary Fig. 4c-d) after at least dozens of cycles. To sum up, for crystal-OSSPSU stator, when the pulse signal voltage is 150 V_{P-P} and the period is less than 5 cycles, and for ceramic-OSSPSU stator, when the pulse signal voltage is 250 V_{P-P} and the period is less than 26 cycles the average horizontal force of the friction tip cannot overcome the static friction of the slider, resulting in the slider remaining in still (which called the deadzone area of the ultrasonic motor)[4,5]. Especially, the measurement of step resolution is in a home-made noise reduction cavity (Supplementary Fig. 9), where the ambient noise in it is around 2nm. Hence, the step displacement cannot be further reduced.

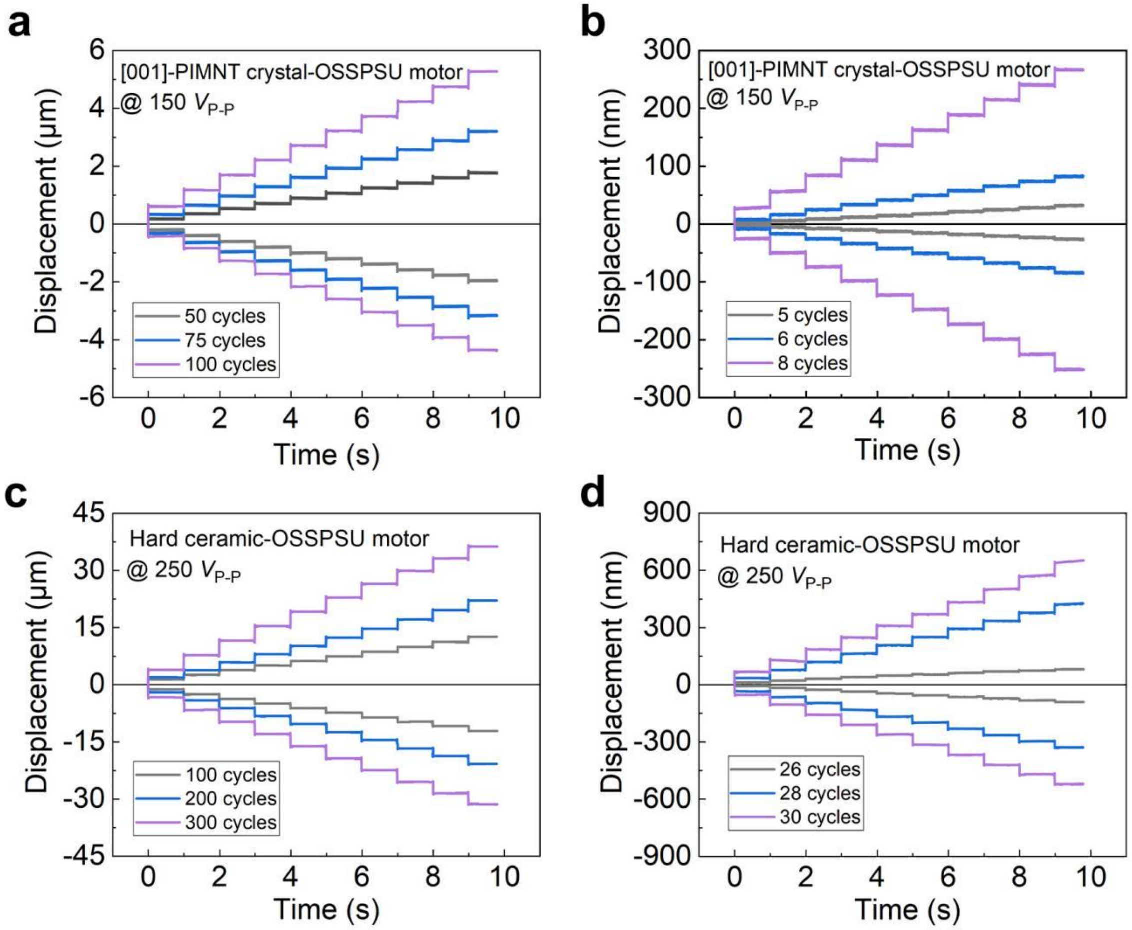

Supplementary Fig. 7 Experimentals of nanostep motion characteristics for piezoelectric OSSPSU motors under the same votage. a-b, The bidirectional motion steps of [001]-PIMNT crystal-OSSPSU motor under different number of period of pulse signals (at 150 $V_{\text{P-P}}$ and 167 kHz). **c-d,** The bidirectional motion steps of hard ceramic-OSSPSU motor under different number of period of pulse signals (at 250 $V_{\text{P-P}}$ and 332 kHz).

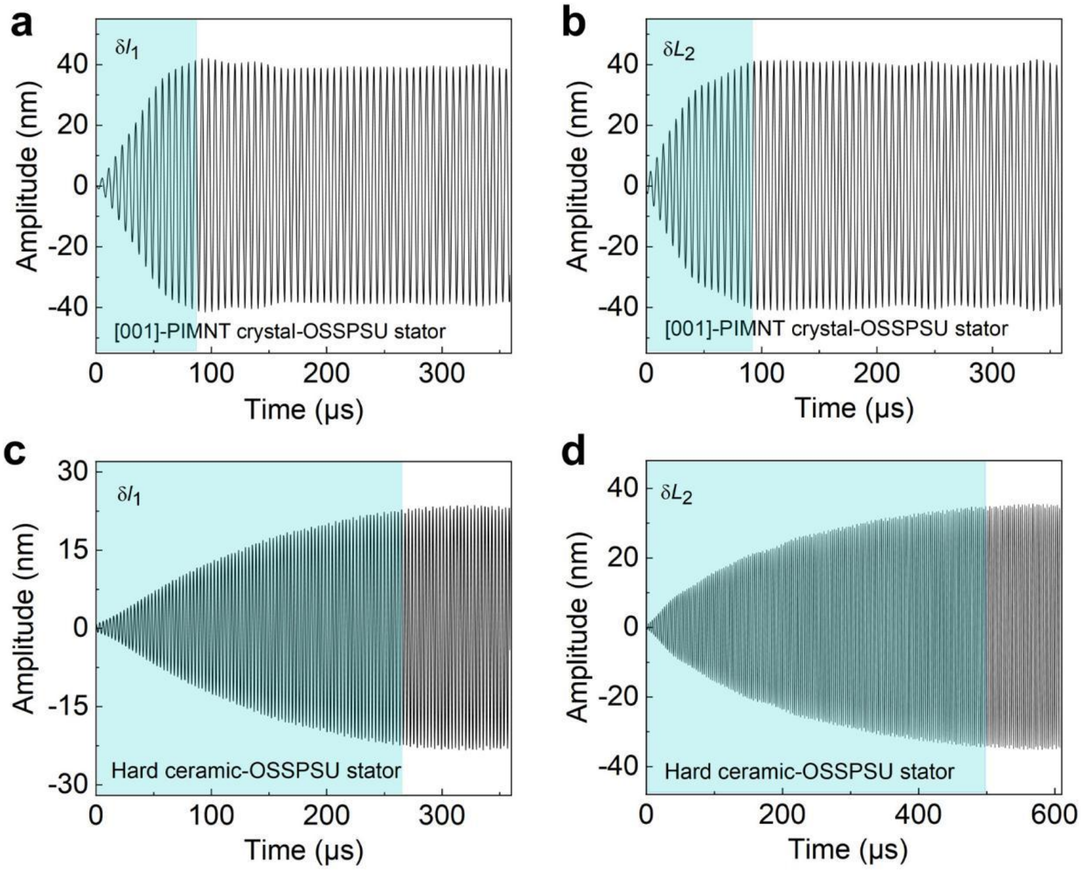

Supplementary Fig. 8 The transient displacement response of the piezoelectric OSSPSU stators. a-b, The transient displacement response of the coupled horizontal (δl_1, along the 1-axis) and coupled vertical (δL_2, along the 2-axis) directions of the friction tips for [001]-PIMNT crystal-OSSPSU stator; **c-d,** hard ceramic-OSSPSU stator under the electric field of 20 V mm^{-1}.

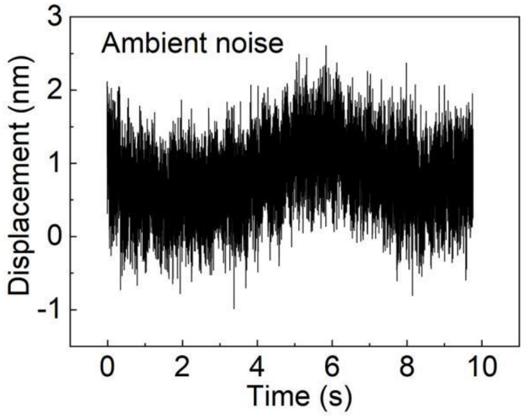

Supplementary Fig. 9 The ambient noise in the noise reduction cavity.

Supplementary Table 2. Comparison of the output performance of the proposed miniature and nanostep OSSPSU motors with other previously reported ultrasonic motors.

Materials	Working state	Volume (mm^3)	Output force in unit volume ($mN\ mm^{-3}$)	Step resolution (nm)	Maximum speed ($mm\ s^{-1}$)	Speed in unit volume ($s^{-1}\cdot mm^{-2}$))	Control method	References
Hard ceramic-OSSPSU	Resonant	21.06	1.09	8	31.3	1.49	Open-loop	This work
[001]-PIMNT crystal-OSSPSU	Resonant	6.89	4.5	3	32.1	4.66	Open-loop	This work
PIMNT crystal	Nonresonant	1800	-	350	3.28	1.82×10^{-3}	Open-loop	7
PIMNT crystal	Resonant	36	6.9	-	35	0.97	Open-loop	8
PZT ceramic	Resonant	15500	3.68	20	450	2.90×10^{-2}	lose-loop	9
$Pb(Sn_{1/3}Nb_{2/3})O_3$-$Pb(Mn_{1/3}Sb_{2/3})O_3$-$Pb(Zr_{1/2}Ti_{1/2})O_3$ ceramic	Resonant	706.5	3.68	200	248	0.35	Open-loop	10
PZT ceramic	Resonant	768	0.079	440	516.3	0.67	Open-loop	11
PZT ceramic	Nonresonant	4960	-	2	0.134	2.70×10^{-5}	Open-loop	12

Supplementary Note 7. The application of the micro linear ultrasonic OSSPSU motor.

To display the excellent output performance of the miniature nanostep piezoelectric OSSPSU motor based on [001]-PIMNT single crystal-OSSPSU stator, a simple auto focus (AF) lens using a commercial slider was designed (as shown in Supplementary Fig. 10). The total mass of the slider and lens is about 1.5 g. Supplementary Fig. 11a shows the relationship (measured at 167 kHz) between the up-and-down motion velocities of AF OSSPSU motor and the applied voltage. It is clearly seen that the maximum speeds of AF OSSPSU motor moving upward and downward are 19.4 mm s^{-1} and 25.4 mm s^{-1}, respectively. This is because the slider and the lens mass act as resistance when moving up and provide power when moving down, resulting in asymmetrical speeds of the two channels. Similarly, the step precision of the two channels of the OSSPSU motor is asymmetrical (Supplementary Fig. 11b). Obviously, the step displacements reduce linearly with the decrease of the cycle of pulse signals, while nd the minimum step is 17 nm, higher than the step shown in Figure 6b. This is because of the inability to use the noise reduction cavity in this test, the ambient noise is relatively large (about 15nm). The results indicate that the miniature nanostep OSSPSU motor based on the [001]-PIMNT single crystal-OSSPSU stator has potential application in lens module, endoscope and other fields.

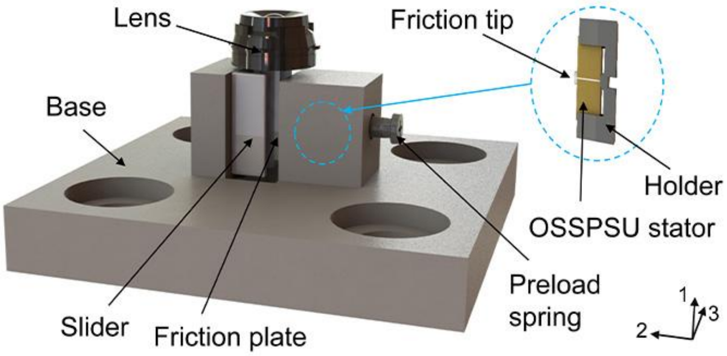

Supplementary Fig. 10 Schematic of the auto focus (AF) OSSPSU motor.

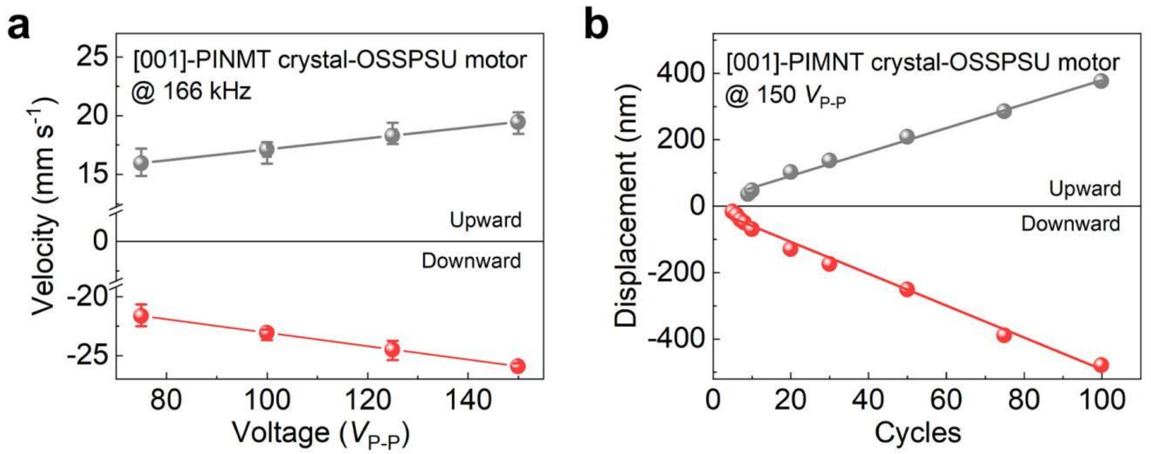

Supplementary Fig. 11 Velocity and nanostep motion performance for the OSSPSU motors. a, The relationship (measured at 167 kHz) between the up-and-down motion velocities of the AF OSSPSU motor and the applied voltage. **b**, The step displacements of the AF OSSPSU motor as a function of the cycle of pulse signals.

Supplementary Note 8. Miniature piezoelectric OSSPSU motor stators and their experimental setup.

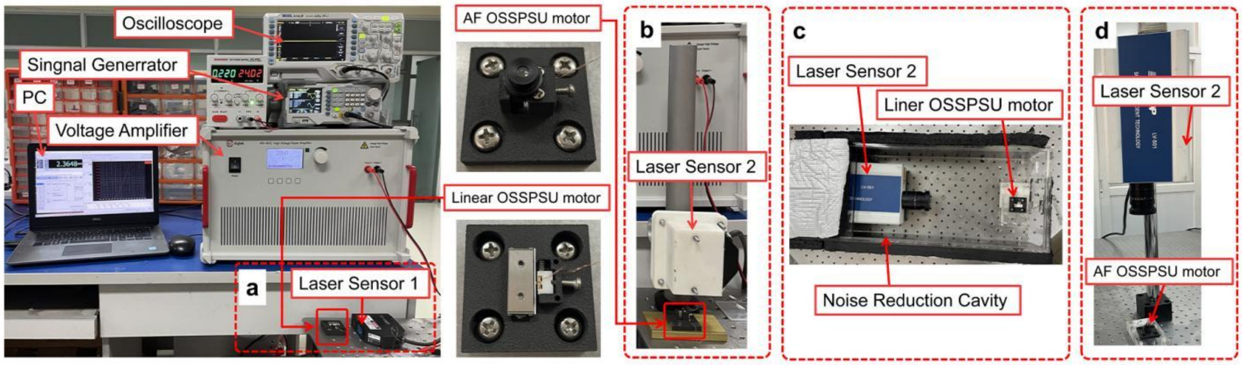

Supplementary Fig. 12. Miniature piezoelectric OSSPSU motor stators and their experimental setup. a, The experimental setup for speed test of the linear OSSPSU motor; **b**, the AF OSSPSU motor; **c**, step displacement test of the linear OSSPSU motor; **d**, the AF OSSPSU motor.

Supplementary References

[1] Han, S. M. et al. Dynamics of transversely vibrating beams using four engineering theories. *J. Sound Vib* **225**, 935-988 (1999).

[2] Chang, S. H. et al. Analysis of methods for determining electromechanical coupling coefficients of piezoelectric elements. *IEEE. T. Ultrason. Ferr* **42**, 630-640 (1995).

[3] Chen, Z. J., Li, X. T., Chen, J. G. & Dong, S. X. A square-plate ultrasonic linear motor operating in two orthogonal first bending modes. *IEEE. T. Ultrason. Ferr* **60**(1), 115-20 (2013).

[4] Xu, Y. H., Feng, Y. J. & Zhang, N. Effect of uniaxial compressive prestress on the electromechanical behavior of lanthanum modified lead zirconate titanate stannate ceramic. *J. Am. Ceram. Soc* **94**, 3863 (2011).

[5] Li, X., Yao, Z. Y., He, Y. G. & Dai, S. C. Modeling and experimental investigation of thermal–mechanical–electric coupling dynamics in a standing wave ultrasonic motor. *Smart Mater. Struct* **26**, 095044 (2017).

[6] Tsai, M.-S., Lee, C.-H. & Hwang, S.-H. Dynamic modeling and analysis of a bimodal ultrasonic motor. *IEEE. T. Ultrason. Ferr* **50**, 245-256 (2003).

[7] Gao, X. Y. et al. A bending-bending mode piezoelectric actuator based on PIN-PMN-PT crystal stacks. *Sensor. Actuat. A-Phys* **331**, 113052 (2021).

[8] Chen, Z. J., Li, X. T., Liu, G. X. & Dong, S. X. A standing wave linear ultrasonic motor operating in in-plane expanding and bending modes. *Rev. Sci. Instrum* **86**, 035002 (2014).

[9] Kim, W.-S., Yun, C.-H.& Lee, S.-K. Nano positioning of a high Power ultrasonic linear motor. *Jpn. J. Appl. Phys* **47**, 5687-5692 (2008).

[10] Xin, X. D. et al. A ring-shaped linear ultrasonic motor based on PSN-PMS-PZT ceramic. *Sensor. Actuat. A-Phys* **309**, 112036 (2020).

[11] Liu, Y. X. et al. Arthropod-metamerism-inspired resonant piezoelectric millirobot. *Adv. Intell. Syst* **3**, 2100015 (2021).

[12] Gao, X. Y. et al. A piezoelectric and electromagnetic dual mechanism multimodal linear actuator for generating macro- and nanomotion. *Research* **2019**, 8232097 (2019).
