# Supplementary material for: Miniaturized electromechanical devices with multi-vibration modes achieved by orderly stacked structure with piezoelectric strain units
Source: Nat Commun. 2022 Nov 2;13:6567. doi: 10.1038/s41467-022-34231-7 (PMC9630418; doi:10.1038/s41467-022-34231-7)
Supplement: Supplementary file 3 — Description of Additional Supplementary Files [file 41467_2022_34231_MOESM3_ESM.pdf]

Description of Additional Supplementary Files

Supplementary Movie 1: The orderly stacked structures based on two piezoelectric strain units and their vibration modes.

Supplementary Movie 2: Working principle GIF and motion video of the ultrasonic OSSPSU motor.
