# Supplementary material for: Miniaturized electromechanical devices with multi-vibration modes achieved by orderly stacked structure with piezoelectric strain units
Source: Nat Commun. 2022 Nov 2;13:6567. doi: 10.1038/s41467-022-34231-7 (PMC9630418; doi:10.1038/s41467-022-34231-7)
Supplement: Supplementary file 6 — Lasing Reporting Summary [file 41467_2022_34231_MOESM6_ESM.pdf]

nature research

Corresponding author(s): Xiangyu Gao, Fei Li

Lasing Reporting Summary

Nature Research wishes to improve the reproducibility of the work that we publish. This form is intended for publication with all accepted papers reporting claims of lasing and provides structure for consistency and transparency in reporting. Some list items might not apply to an individual manuscript, but all fields must be completed for clarity.

For further information on Nature Research policies, including our data availability policy, see Authors & Referees.

ü Experimental design

Please check: are the following details reported in the manuscript?

1. Threshold

Plots of device output power versus pump power over a wide range of values indicating a clear threshold
☐ Yes
☒ No
No lasing was used in the manuscript.

2. Linewidth narrowing

Plots of spectral power density for the emission at pump powers below, around, and above the lasing threshold, indicating a clear linewidth narrowing at threshold
☐ Yes
☒ No
No lasing was used in the manuscript.

Resolution of the spectrometer used to make spectral measurements
☐ Yes
☒ No
No lasing was used in the manuscript.

3. Coherent emission

Measurements of the coherence and/or polarization of the emission
☐ Yes
☒ No
No lasing was used in the manuscript.

4. Beam spatial profile

Image and/or measurement of the spatial shape and profile of the emission, showing a well-defined beam above threshold
☐ Yes
☒ No
No lasing was used in the manuscript.

5. Operating conditions

Description of the laser and pumping conditions
Continuous-wave, pulsed, temperature of operation
☐ Yes
☒ No
No lasing was used in the manuscript.

Threshold values provided as density values (e.g. W cm^{-2} or J cm^{-2}) taking into account the area of the device
☐ Yes
☒ No
No lasing was used in the manuscript.

6. Alternative explanations

Reasoning as to why alternative explanations have been ruled out as responsible for the emission characteristics
e.g. amplified spontaneous, directional scattering; modification of fluorescence spectrum by the cavity
☐ Yes
☒ No
No lasing was used in the manuscript.

7. Theoretical analysis

Theoretical analysis that ensures that the experimental values measured are realistic and reasonable
e.g. laser threshold, linewidth, cavity gain-loss, efficiency
☐ Yes
☒ No
No lasing was used in the manuscript.

8. Statistics

Number of devices fabricated and tested
☐ Yes
☒ No
No lasing was used in the manuscript.

Statistical analysis of the device performance and lifetime (time to failure)
☐ Yes
☒ No
No lasing was used in the manuscript.
